# The Reliability of Two- and Three-Dimensional Cephalometric Measurements: A CBCT Study

**DOI:** 10.3390/diagnostics11122292

**Published:** 2021-12-07

**Authors:** Chenshuang Li, Hellen Teixeira, Nipul Tanna, Zhong Zheng, Stephanie Hsiang Yi Chen, Min Zou, Chun-Hsi Chung

**Affiliations:** 1 Department of Orthodontics, School of Dental Medicine, University of Pennsylvania, Philadelphia, PA 19104, USA; hellen@dental.upenn.edu (H.T.); nipul77@upenn.edu (N.T.); stephhc@upenn.edu (S.H.Y.C.); 2 David Geffen School of Medicine, University of California, Los Angeles, CA 90095, USA; zzheng@dentistry.ucla.edu; 3 School of Dentistry, University of California, Los Angeles, CA 90095, USA; 4 Key Laboratory of Shannxi Province for Craniofacial Precision Medicine Research, College of Stomatology, Xi’an Jiaotong University, 98 XiWu Road, Xi’an 710004, China; zoumin@mail.xjtu.edu.cn; 5 Clinical Research Center of Shannxi Province for Dental and Maxillofacial Diseases, College of Stomatology, Xi’an Jiaotong University, 98 XiWu Road, Xi’an 710004, China; 6 Department of Orthodontics, College of Stomatology, Xi’an Jiaotong University, 98 XiWu Road, Xi’an 710004, China

**Keywords:** cephalometric analysis, two-dimensional, three-dimensional, CBCT, orthodontics

## Abstract

Cephalometry is a standard diagnostic tool in orthodontic and orthognathic surgery fields. However, built-in magnification from the cephalometric machine produces double images from left- and right-side craniofacial structures on the film, which poses difficulty for accurate cephalometric tracing and measurements. The cone-beam computed tomography (CBCT) images not only allow three-dimensional (3D) analysis, but also enable the extraction of two-dimensional (2D) images without magnification. To evaluate the most reliable cephalometric analysis method, we extracted 2D lateral cephalometric images with and without magnification from twenty full-cranium CBCT datasets; images were extracted with magnification to mimic traditional lateral cephalograms. Cephalometric tracings were performed on the two types of extracted 2D lateral cephalograms and on the reconstructed 3D full cranium images by two examiners. The intra- and inter-examiner intraclass correlation coefficients (ICC) were compared between linear and angular parameters, as well as between CBCT datasets of adults and children. Our results showed that overall, tracing on 2D cephalometric images without magnification increased intra- and inter-examiner reliability, while 3D tracing reduced inter-examiner reliability. Angular parameters and children’s images had the lowest inter- and intra-examiner ICCs compared with adult samples and linear parameters. In summary, using lateral cephalograms extracted from CBCT without magnification for tracing/analysis increased reliability. Special attention is needed when analyzing young patients’ images and measuring angular parameters.

## 1. Introduction

Dr. Broadbent first established reproducible head positioning of the cephalostat in 1930 [1], which set a precedent for using lateral cephalometric radiographs in orthodontics. When Dr. Brodie used cephalometric X-rays to investigate craniofacial growth factors affecting orthodontic treatment in 1941 [2], and Dr. Margolis evaluated the relationship between incisor inclinations and various craniofacial factors in 1943 [3], cephalometric analysis started to evolve. In 1948, Dr. Downs developed the first cephalometric analysis method [4]. Since then, multiple methods have been established, and lateral cephalometric X-rays have been used as the standardized, reproducible tool for orthodontic diagnosis and treatment planning [5].

Initially, lateral cephalograms were developed on film, and analyses were performed manually using acetate tracing paper over a lighted view box. Despite having served the medical community well for many years, this conventional screen-film radiology technique required stringent exposure factors to produce a diagnostic-quality radiograph. Slight underexposure or overexposure could yield an unacceptable image, which significantly increased tracing difficulty [6]. Another drawback of conventional film was that errors during exposure could not be remedied. For example, improper head orientation would cause distortion of craniofacial structures, which negatively affected clinical diagnosis [7]. Retaking radiographs due to errors led to an increase in radiation exposure to the technician and patient, increased cost of the examination, and required additional technician and clinician time [8]. In addition, the procedure required to develop film delayed clinical diagnosis and increased cost [9]. Notably, due to the divergent pattern of the x-ray beam and the distance between the subject and image-receptor in the cephalometric machine system [10], built-in magnification enlarged the left and right craniofacial structures at different ratios. Thus, even with proper exposure and head positioning, double images were present on the film, which made accurate cephalometric tracing difficult and led to tracing errors among orthodontic residents and clinicians [11]. More importantly, the magnification of X-rays was not consistent across different machines [12], which limited the ability to compare study results among different research groups.

The introduction of digitalized radiographs not only eliminates the time-consuming steps of film development, but also allows image contrast adjustment, which mitigates exposure problems and reduces the likelihood of repeated exposure [13]. More importantly, digitalized radiographs enable digital cephalometric analysis, which make tracing and diagnosis faster and easier, without reducing accuracy [14,15,16]. However, digitalized radiograph systems do not solve the problems caused by improper head orientation, or the magnification inherent to 2D imaging. Furthermore, magnification ratios among different digital cephalometric machines remain inconsistent [17,18], which leaves comparison among studies conducted with different machines not completely reliable.

Over the past decade, cone-beam computed tomography (CBCT) systems have been broadly adapted in orthodontic and orthognathic surgery fields, which allow three-dimensional analysis without magnification and distortion [5]. With radiation exposure and cost significantly reduced since the initial introduction of CBCT, 3D imaging has become more widely used to replace several 2D X-rays images for diagnosis and progress evaluation [19]. Although the radiation doses from dental CBCT exams are generally lower than other CT exams, dental CBCT exams typically deliver more radiation than conventional dental X-ray exams [20]. Concerns about radiation exposure are greater for younger patients because they are more sensitive to radiation, and they have a longer lifetime for ill-effects to develop. This consideration is important in the orthodontic field, as the majority of orthodontic patients are children and teenagers, and multiple routine radiation exposures are planned during orthodontic treatment to evaluate growth and development as well as treatment progress [21]. A recent study estimated the cancer risk from CBCT in orthodontic patients, and reported that the risk of exposure-induced death values in the 10-year-old subjects were approximately double those in the 30-year-old subjects for all cancers, especially in breast cancer of females [22]. Thus, radiation protection protocols should be rigorously followed when prescribing radiological images to patients.

Since 2D cephalometric tracing and superimposition are still the standard methods for orthodontic and orthopedic treatment evaluation [23], great efforts have been dedicated to comparing 2D and 3D cephalometric analysis globally [19,24,25,26]. As several 2D cephalometric analysis landmarks are artificial structures defined from overlapped sagittal plane images, the same landmarks are harder to identify in 3D reconstructed images [19,27]. Thus, some clinicians and researchers suggest establishing new landmarks on CBCTs, and setting up a new 3D cephalometric tracing and analysis system [19]. There is no doubt that 3D cephalometric tracing and analysis can provide more information for comprehensive clinical evaluation and diagnosis, but an inevitable challenge will be comparing the results of new studies with the well-validated and accepted knowledge in the orthodontic field.

CBCT not only allows for 3D evaluation of craniofacial structures, but also enables the generation of 2D images with or without magnification. Although great efforts have gone into the comparison of conventional 2D and 3D tracings, no evaluation has been done using 2D lateral cephalometric images extracted from CBCTs without magnification. Additionally, whether patients’ maturation stages affect tracing reliability has not been assessed. Thus, in the current study, we compared the intra- and inter-examiner reliability of 2D cephalometric analysis conducted with CBCT extracted lateral cephalometric images with magnification, which mimic traditional lateral cephalograms taken with proper head position; 2D cephalometric analysis conducted with CBCT extracted lateral cephalometric images without magnification; and 3D cephalometric analysis conducted with CBCT reconstructed images. The intra- and inter-examiner correlations were calculated and compared between linear and angular parameters, as well as between adults’ and children’s CBCT datasets. Overall, the current study aims to provide more information about the factors influencing tracing reliability, which could potentially improve clinical data analysis and comparison.

## 2. Materials and Methods

**Figure 1:**
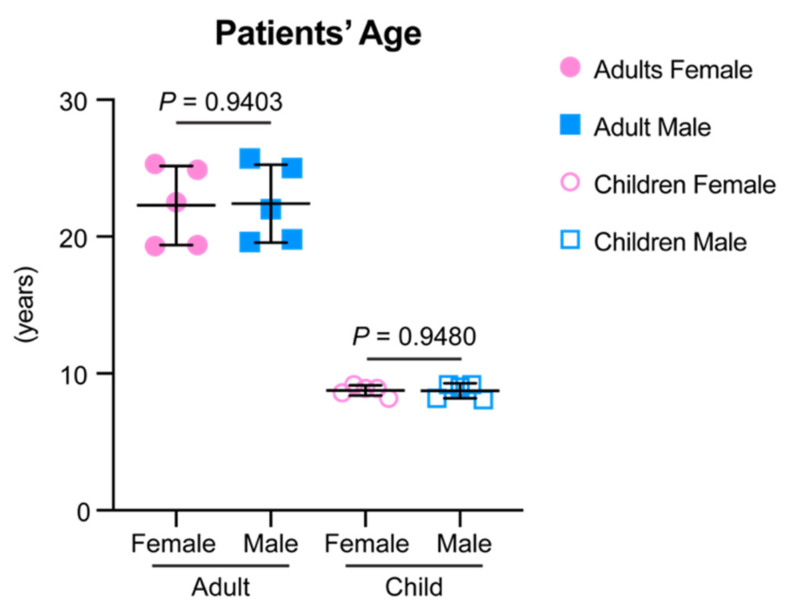
The age information of the samples included in the current study. Data are presented as raw data overlapped with mean ± standard deviation (SD). Two-tailed *t*-test was used for statistical analysis. No statistically significant difference was detected between genders for each age group.

CBCT scans for this study were derived from a pre-existing clinical database of pre-orthodontic treatment records, and the study protocol was approved by the institution review board (protocol #848424). No additional radiographic images were taken for study purposes. Images were taken with a voxel size of 0.400 × 0.400 × 0.400 mm^3^. CBCTs from 20 patients without craniofacial syndromes or large facial asymmetry were included. Among these 20 patients, 10 were adults with permanent dentition (five females [mean age 22.4 years, range 19.3 years–25.3 years], five males [mean age 22.4 years, range 19.6 years–25.7 years]), and 10 were children with early mixed dentition (five females [mean age 8.8 years, range 8.2 years–9.2 years], five males [mean age 8.7 years, range 8.1 years–9.2 years]) (Figure 1).

**Figure 2:**
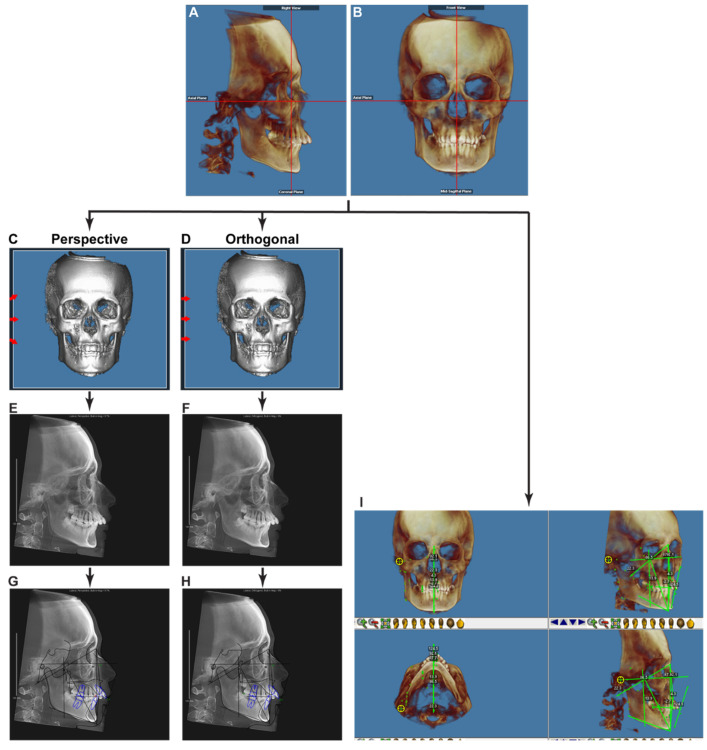
The imaging processing flow chart of the current study. (**A**,**B**) The orientation of a CBCT image. (**C**,**D**) Under the “X-ray Building preferences” option, “Perspective” was selected to create an X-Ray with the measured distortion and warping effects mimicking a traditional X-Ray (**C**), and “Orthogonal” was selected to create a non-distorted X-Ray (**D**). The red arrows represent the direction of X-Rays beams simulated by computer calculation. (**E**,**F**) The generated lateral cephalometric X-Rays with (**E**) or without (**F**) magnification and distortion. Note the two mandibular posterior borders visible in (**E**), but not in (**F**). (**G**,**H**) Digital cephalometric tracing and analysis were performed with the extracted 2D lateral cephalometric images. (**I**) Tracings with 3D reconstructed images were performed by using the “Digitize/Measurement” function in Dolphin 3D.

All CBCT DICOM files were imported into Dolphin 3D software (Dolphin Imaging; version 11.95 Premium, Chatsworth, CA, USA) and oriented using the Frankfort plane as the horizontal plane (Figure 2A). The orientation was adjusted axially, so that lateral borders of the orbits from a lateral view overlapped each other (Figure 2A), and coronally so that inferior borders of both orbits sat on the same plane from a frontal view (Figure 2B). Utilizing the “Build X-Rays Tool” in Dolphin 3D, lateral cephalometric X-Rays were created using both sides of the volume. Under the “X-ray Building preferences” option, “Perspective” was selected to create an X-Ray with the measured distortion and warping effects of a traditional X-Ray (Figure 2C,E), and “Orthogonal” was selected to create a non-distorted X-Ray (Figure 2D,F). According to “Dolphin Imaging User’s Guide (Version 11.95)”, the settings for “Perspective” were set as follows to match with the Bolton-Broadbent dimensions:

- Fictitious Magnification Factor: 9.7%;
- Emitter to Patient’s Mid Plane (Distance in millimeters from the emitter to the patient’s mid-plane to use when generating X-rays using perspective projection): 1550 mm;
- Mid-Plane to Film (Distance in millimeters from patient’s mid-plane to the film to use when generating X-Rays using perspective projection): 150 mm.

The generated 2D lateral cephalometric X-Rays were traced in the “Digitize” function module in Dolphin Imaging (Figure 2G,H). At the same time, tracings of 3D reconstructed images were performed by using the “Digitize/Measurement” function in Dolphin 3D (Figure 2I). Ricketts (comprehensive) [28], Steiner + Wits [29,30], Sassouni+ [31], and McNamara [32] cephalometric analysis systems were used. After removing repeated parameters, 63 (28 linear, 35 angular) parameters were used for further analysis.

Image extraction and tracing were performed by two examiners, both American Board of Orthodontics certified clinicians and full-time educators in an academic institution. Calibration between the two examiners was performed twice before formal analysis. The two examiners performed cephalometric analysis separately, and each image was analyzed twice by each examiner at least one month apart.

The values from two sets of cephalometric analyses were used to calculate the intra-examiner intraclass correlation coefficient (ICC), and the average value of two sets of cephalometric analyses from each examiner was used to calculate the inter-examiner ICC. The intra- and inter-examiner ICCs of each parameter were calculated utilizing the IBM SPSS software (Statistical Package for Social Sciences version 26.0, Chicago, IL, USA), and compared between linear and angular parameters, as well as between adults’ and children’s CBCT datasets. A Shapiro-Wilk normality test was performed by OriginPro 8 (Origin Lab Corp., Northampton, MA, USA). A two-tailed *t*-test was used for statistical analysis of the patients’ age comparison. Since some of the ICC data did not pass the normal distribution test, all ICC data were presented with a violin plot. A Wilcoxon matched-pairs signed-rank test was used for statistical analysis of the overall comparison of intra- and inter-examiner ICCs, and the comparison within each type of parameter. A Mann-Whitney *U* test was used for statistical analysis for the comparison between the two types (linear and angular) of parameters. For all data presented in this manuscript, *p* < 0.05 (*) was considered a suggestive difference, while *p* < 0.005 (**) was recognized as a statistically significant difference based on a recent recommendation [33].

## 3. Results

### 3.1. Comparisons of the Intra- and Inter-Examiner Reliability among the Tracings of Different Images

We first compared the intra- and inter-examiner ICCs for all measurement parameters with 20 samples together. The intra-examiner ICCs for each parameter are listed in Table 1, and the inter-examiner ICCs are listed in Table 2.

**Figure 3:**
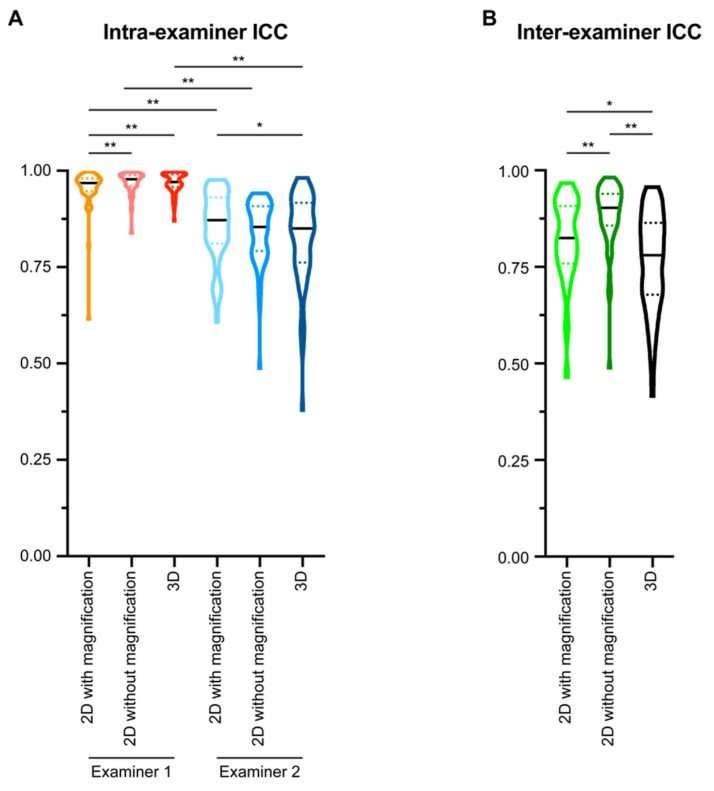
The intra- and inter-examiner intraclass correlation coefficient (ICC) of all measurement parameters. (**A**) The intra-examiner ICCs were calculated for each examiner based on two sets of cephalometric tracing measurements performed with at least a one-month interval. (**B**) The inter-examiner ICC of the two examiners was calculated by comparing the mean value of the two sets of cephalometric tracing measurements from each examiner. All data presented with violin plots. The solid black line in each violin plot indicates the median, and the colored dotted lines in each violin plot indicates the quartiles. A Wilcoxon matched-pairs signed-rank test was used for statistical analysis. *: *p* < 0.05; **: *p* < 0.005.

For examiner 1 (Figure 3A), the median of the ICCs in the 2D tracings of extracted lateral cephalometric images with magnification was 0.968. Removing magnification could statistically significantly increase the median of the ICCs to 0.978 (*p* < 0.001). When evaluating the 3D tracings of examiner 1, the median of the ICCs was 0.971, which was statistically significantly higher than that of 2D tracings with magnification (*p* = 0.002), but not different to that of 2D tracings without magnification.

For examiner 2 (Figure 3A), the median of the ICCs in the 2D tracings with magnification was 0.872, and the median of the ICCs in the 2D tracings without magnification was 0.854. No statistical significance was detected when comparing the intra-examiner ICC of examiner 2 for the two types of 2D tracings. For the 3D tracings of examiner 2, the median of the ICCs was 0.850. There is no statistical significance between the intra-examiner ICC of examiner 2 in the 2D tracings without magnification and 3D tracing, but the 3D tracing had suggestively significantly lower intra-examiner ICC than the 2D tracings with magnification (*p* = 0.0461).

We then evaluated the inter-examiner reliability for each type of cephalometric analysis method (Figure 3B). In the 2D tracings with magnification, the median of the ICCs was 0.824. In the 2D tracings without magnification, the median of the inter-examiner ICCs was 0.903. In the 3D tracings, the median of the inter-examiner ICCs was 0.780. Comparison among the three types of cephalometric analysis methods showed that 2D tracings without magnification had the highest inter-examiner reliability (*p* < 0.0001), in the range of excellent [34]. Both 2D tracings with magnification and 3D tracings had good inter-examiner ICCs [34], while 2D tracings with magnification suggested a higher inter-examiner ICC than that of 3D tracings (*p* = 0.0066).

### 3.2. Comparison between Linear and Angular Parameters

Previous studies showed that different cephalometric tracing methods may affect the measured results of linear and angular parameters differently [18,35,36]. Thus, in the current study, we compared the intra- and inter-examiner reliabilities of linear and angular parameters for all three types of tracing methods.

**Figure 4:**
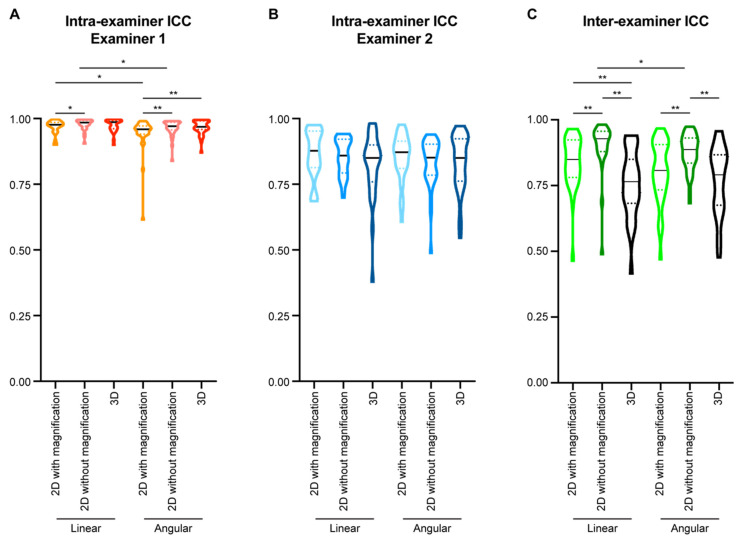
Comparison of intra- and inter-examiner intraclass correlation coefficient (ICC) in different measurement parameters. (**A**,**B**) The intra-examiner ICCs of examiner 1 (**A**) and examiner 2 (**B**) were calculated for each examiner based on two sets of cephalometric tracing measurements performed with at least a one-month interval. (**C**) The inter-examiner ICC of the two examiners was calculated by comparing the mean value of the two sets of cephalometric tracing measurements from each examiner. All data presented with violin plots. The solid black line in each violin plot indicates the median, and the colored dotted lines in each violin plot indicates the quartiles. Wilcoxon matched-pairs signed-rank test was used for statistical analysis for the comparison within each type of the parameters, and Mann-Whitney *U* test was used for statistical analysis for the comparison between two types of the parameters. *: *p* < 0.05; **: *p* < 0.005.

For examiner 1 (Figure 4A), the intra-examiner ICC of linear parameters was suggestively higher than that of angular parameters (*p* = 0.0052) in the 2D tracings of extracted lateral cephalometric images with magnification. Tracing on 2D images without magnification could improve the intra-examiner ICCs for both linear (*p* = 0.0210) and angular parameters (*p* = 0.0017), while linear parameters still had higher intra-examiner ICCs than angular parameters (*p* = 0.0146). 3D tracings did not alter the intra-examiner ICCs of the linear parameters when compared with the two types of 2D tracings, but significantly increased the intra-examiner ICCs of the angular parameters when compared with the 2D tracings with magnification (*p* = 0.0029). However, there was no statistical significance when comparing angular and linear parameters in 3D tracings.

For examiner 2 (Figure 4B), there was no statistical significance between linear and angular parameters. Changing tracing methods did not alter the intra-examiner ICCs for both linear and angular parameters.

Moving to the inter-examiner reliability (Figure 4C), in the 2D tracings with magnification, there was no statistical significance when comparing the inter-examiner ICC of linear and angular parameters. In the 2D tracings without magnification, linear parameters had suggestively higher inter-examiner ICCs than angular parameters (*p* = 0.0240). 2D tracings without magnification had statistically significantly higher inter-examiner ICCs for both linear and angular parameters than 2D tracings with magnification (*p* < 0.0001). 3D tracings influenced the inter-examiner reliability differently: it decreased the inter-examiner ICC of the linear parameters (*p* = 0.0017), while it did not affect that of the angular parameters when compared to 2D tracings with magnification. 3D tracings had statistically significantly lower inter-examiner ICCs for both linear and angular ICCs than 2D tracings without magnification (*p* < 0.0001).

### 3.3. Comparison between the Images from Adult Patients and the Images from Children Patients

**Figure 5:**
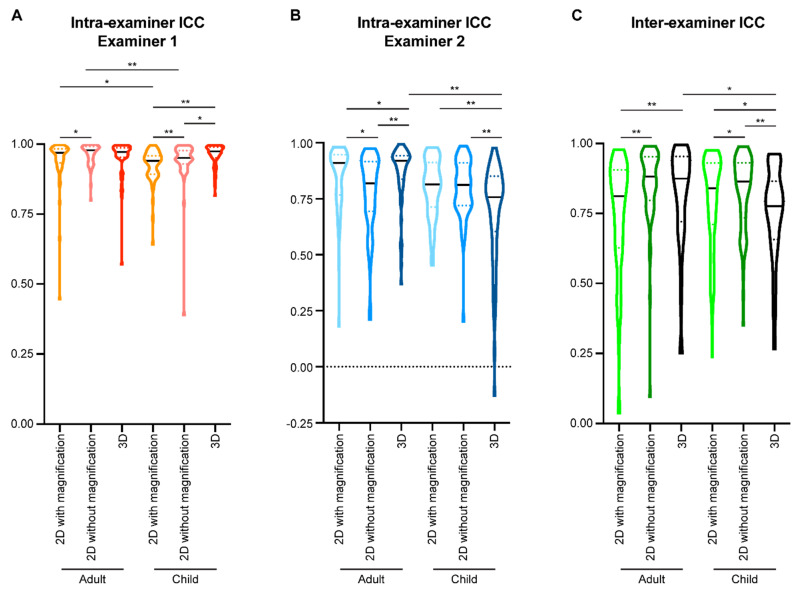
Comparison of intra- and inter-examiner intraclass correlation coefficient (ICC) of all measurement parameters for the radiographic images from different age groups of the patients. (**A**,**B**) The intra-examiner ICCs of examiner 1 (**A**) and examiner 2 (**B**) were calculated for each examiner based on two sets of cephalometric tracing measurements performed with at least a one-month interval. (**C**) The inter-examiner ICC of the two examiners was calculated by comparing the mean value of the two sets of cephalometric tracing measurements from each examiner. All data presented with violin plots. The solid black line in each violin plot indicates the median, and the colored dotted lines in each violin plot indicates the quartiles. Wilcoxon matched-pairs signed-rank test was used for statistical analysis. *: *p* < 0.05; **: *p* < 0.005.

In the current study, we included 10 young patients in early mixed dentition to represent the patient population seeking early orthodontic intervention, and 10 adult patients in permanent dentition to represent patients needing comprehensive orthodontic treatment. The intra-examiner ICCs for each parameter in different age groups were listed in Supplemental Tables S1 and S2, and the inter-examiner ICCs were listed in Supplemental Table S3. For examiner 1 (Figure 5A), in the 2D tracings of extracted lateral cephalometric images with magnification, the intra-examiner ICC for images from adult patients was higher than that from children patients (*p* = 0.0059). Tracing 2D images without magnification could improve the intra-examiner ICCs for both adults (*p* = 0.0247) and children (*p* = 0.0002), while tracings from adult patients still had higher intra-examiner ICCs than that of children (*p* = 0.0015). 3D tracings could further improve the intra-examiner ICCs of the tracings of images from children, but not for that of adults. There was no statistical significance when comparing the intra-examiner ICCs of 3D tracings with images from adult and children patients.

For examiner 2 (Figure 5B), there was no statistical significance when comparing the intra-examiner ICCs of 2D tracings with images from adult and children patients. Interestingly, 3D tracings could increase the intra-examiner ICCs of images from adult patients, but decreased the intra-examiner ICCs of images from children patients.

When looking at the inter-examiner reliability (Figure 5C), there was no statistical significance when comparing the inter-examiner ICCs of 2D tracings with images from adult and children patients. In both age groups, 2D tracings without magnification had higher ICCs than 2D tracings with magnification. 3D tracings had higher ICC than 2D tracings with magnification in the adult group (*p* = 0.0035), but lower ICC than 2D tracings with magnification in the children group (*p* = 0.0348).

### 3.4. Combined Influent Effects of Parameter Types and Patients’ Age on Tracing Reliabilities

Since both parameter types and patients’ age affected the reliability of cephalometric analysis as mentioned above, we performed a more detailed comparison with the consideration of both factors in each type of tracing method.

**Figure 6:**
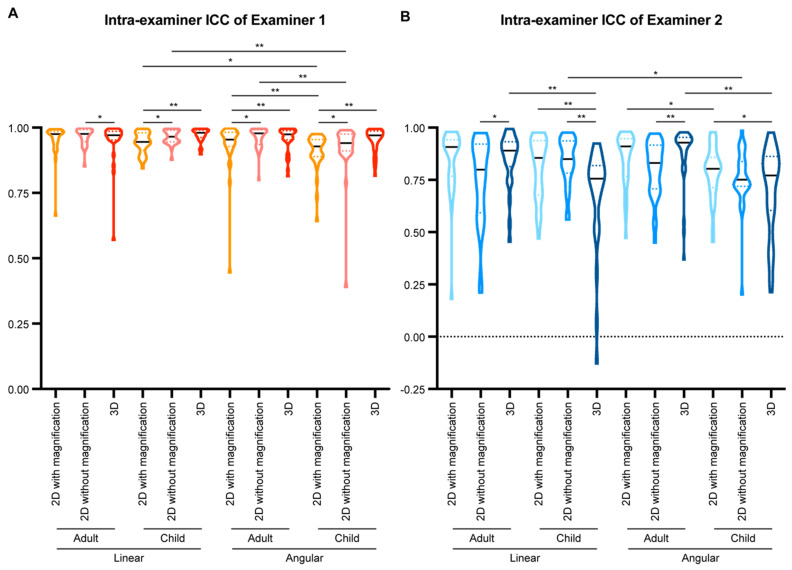
Comparison of intra-examiner intraclass correlation coefficient (ICC) of different measurement parameters for the radiographic images from different patient age groups. (**A**,**B**) The intra-examiner ICCs of examiner 1 (**A**) and examiner 2 (**B**) were calculated for each examiner based on two sets of cephalometric tracing measurements performed with at least a one-month interval. All data presented with violin plots. The solid black line in each violin plot indicates the median, and the colored dotted lines in each violin plot indicates the quartiles. Wilcoxon matched-pairs signed-rank test was used for statistical analysis for the comparison within each type of the parameters, and Mann-Whitney *U* test was used for statistical analysis for the comparison between two types of the parameters. *: *p* < 0.05; **: *p* < 0.005.

For examiner 1 (Figure 6A), in the 2D tracings of extracted lateral cephalometric images with magnification, there was no statistical significance between linear and angular parameters of images from adult patients. However, statistical significance was detected between linear and angular parameters of images from children patients (*p* = 0.0024). In addition, angular parameters of images from children patients had significantly lower intra-examiner ICCs than those from adult patients (*p* = 0.0383). Thus, angular parameters of images from children patients had the lowest intra-examiner ICCs in the 2D tracings with magnification. The same trends can also be observed in the 2D tracings without magnification. In the 3D tracings, no statistical significance was detected among groups. When comparing among different types of tracings for examiner 1, angular parameters of images from children in 2D tracings with magnification had the lowest intra-examiner ICCs.

Similarly, for examiner 2 (Figure 6B), in the 2D tracings with magnification, there was no statistical significance between linear and angular parameters of images from adult patients, and statistical significance was detected between linear and angular parameters of images from children patients (*p* = 0.0118). In the 2D tracings without magnification, examiner 2 also had the lowest intra-examiner ICC with angular parameters of images from children patients. In the 3D tracings, both linear and angular parameters of images from children patients had lower intra-examiner ICCs than did images from adult patients.

**Figure 7:**
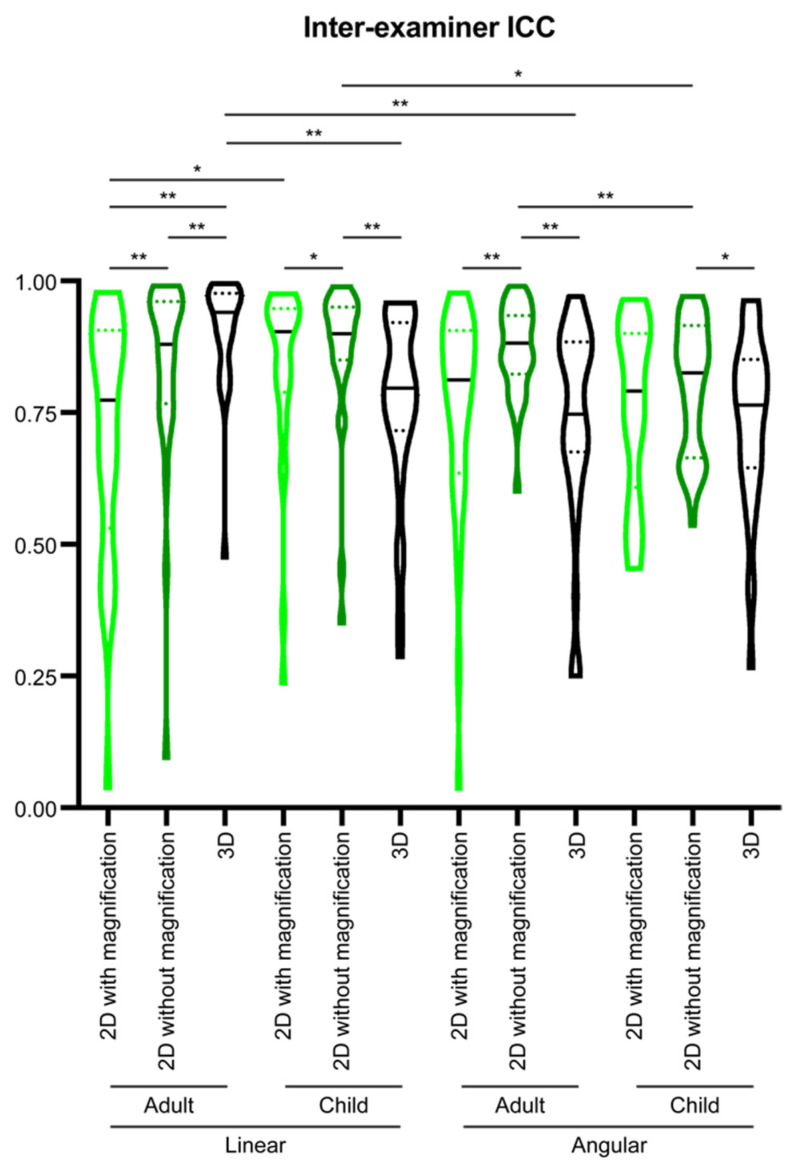
Comparison of inter-examiner intraclass correlation coefficient (ICC) of different measurement parameters for the radiographic images from different patient age groups. The inter-examiner ICC of the two examiners was calculated by comparing the mean value of the two sets of cephalometric tracing measurements from each examiner. All data presented with violin plots. The solid black line in each violin plot indicates the median, and the colored dotted lines in each violin plot indicates the quartiles. Wilcoxon matched-pairs signed-rank test was used for statistical analysis for the comparison within each type of the parameters, and Mann-Whitney *U* test was used for statistical analysis for the comparison between two types of the parameters. *: *p* < 0.05; **: *p* < 0.005.

For inter-examiner reliabilities (Figure 7), linear parameters for the images from children patients had suggestively higher inter-examiner ICC than did the images from adult patients (*p* = 0.0445), while no statistical significance was detected for other comparisons in the 2D tracings with magnification. 2D tracings without magnification had higher inter-examiner ICCs than 2D tracings with magnification in linear parameters of images from both adults and children, and in angular parameters of images from adult patients, but not in angular parameters of images from children patients. In fact, angular parameters of images from children patients had the lowest inter-examiner ICCs when compared to other groups in the 2D tracings without magnification. 3D tracing could only increase the inter-examiner ICCs of linear parameters of images from adult patients, and the ICCs were significantly higher than in linear parameters of images from children patients and in angular parameters of images from both age groups in 3D tracings.

## 4. Discussion

In this study, we compared three types of digital tracings and evaluated the influence of parameter types and patients’ age on the intra- and inter-examiner reliabilities.

When comparing the intra-examiner ICCs for each type of cephalometric analysis between the two examiners, examiner 1 had statistically significantly higher ICCs than examiner 2 (*p* < 0.0001, Figure 3A), which may be because examiner 1 has more imaging analysis experience. However, both examiners had ICC medians for all three types of cephalometric analysis methods higher than 0.75, indicating that all methods had good (between 0.75 and 0.9) to excellent (greater than 0.9) intra-examiner reliability [34]. The ICCs are similar to or higher than those reported in previous publications by other groups [37,38,39,40], demonstrating consistency and accuracy of the present study.

As expected, reducing double images on lateral cephalometric X-rays by eliminating magnification and distortion significantly improved the inter-examiner reliability from good (0.8240) to excellent (0.9030) (Figure 3B). Interestingly, 3D tracing had the lowest inter-examiner ICC among all three types of tracing methods, even though the median ICC was still in the range of good (0.7800) (Figure 3B). Both examiners experienced difficulty identifying certain landmarks during 3D tracings, such as orbitale, porion, DC point, and PT point. These landmarks are formed by overlapping craniofacial structures from different sagittal layers in 2D images. In addition, both examiners had low confidence identifying incisor root tips during 3D tracings. These difficulties are consistent with those encountered in previous studies where the authors compared landmark identification errors on cone-beam computed tomography and conventional digital cephalograms. They found that gonion, condylion, and porion were located on flat or curved surfaces and thus difficult to precisely reference/define on CBCT images [27]. Additionally, certain locations with lower densities, such as the mandibular incisor apex, will have high measurement errors because they could not be visualized with 3D reconstruction [27]. The current study further supports the idea that traditional 2D landmarks do not completely map to 3D tracings, and new landmarks need to be identified in three axial planes to establish a more reliable 3D tracing system [19,41].

When looking at each parameter in detail, cranial deflection angle consistently had low intra- and inter-examiner ICCs in 2D tracings. Thus, the current study suggests that caution is needed while interpreting this measurement clinically.

Both linear and angular parameters have been evaluated by comparing 2D and 3D tracings, with more focus on angular parameters. However, the conclusion on whether angular parameters have similar ICCs in 2D and 3D tracing is controversial [37,42,43]. In the current study, no dramatic difference was found in the intra- or inter- examiner ICCs between linear and angular parameters. Using 2D tracings of extracted lateral cephalometric images without magnification could improve the intra- and inter-examiner ICCs for both linear and angular parameters when compared to 2D tracings with magnification. 3D tracings had low inter-examiner ICCs for linear and angular parameters relative to 2D tracings, but there was no difference between linear and angular parameters within 3D tracings (Figure 4). Thus, linear parameters are more reliable than angular parameters in 2D tracings.

Unerupted permanent teeth overlap with maxillary and mandibular alveolar bone, which increases difficulty when tracing lateral cephalometric X-rays of children with mixed dentition. The low bone density of the children compared to adults may also add difficulty to landmark identification during the cephalometric analysis of young patients. Thus, in the current study, we evaluated whether there is any difference in the intra- and inter-examiner reliabilities of cephalometric analysis on X-rays from patients of different ages and dentition types. To the best of our knowledge, this is the first study making this comparison. For intra-examiner reliability, a small but statistically significant difference was found between the adult (ICC median 0.9690) and children groups (ICC median 0.9400) in the 2D tracings with magnification in one examiner (Figure 5A). Removing the magnification and distortion from the 2D X-rays could improve both intra- and inter-examiner reliability of the cephalometric analysis (Figure 5), but the adult group still had higher intra-examiner ICC than the children group with the same examiner (Figure 5A). For the 3D tracing, opposite trends were observed with different age groups: compared to the 2D tracings with magnification, 3D tracing could increase the intra- (Figure 5B) and inter-examiner ICCs (Figure 5C) in the adult group, but decrease the intra-examiner ICC with the examiner who has less experience with 3D imaging analysis in the children group (Figure 5B), and further decrease the inter-examiner ICCs of the children group (Figure 5C). With all the medians of ICCs for each type of cephalometric analysis in both age groups higher than 0.75, all tracing methods were reliable for both children and adult patients. However, the current study suggests clinical caution is needed while evaluating the images from children patients.

With the consideration of tracing methods, parameter types, and patients’ ages, a detailed comparison was performed. We found that angular measurements of the images from children patients had the lowest intra-examiner reliabilities for both examiners in all three types of cephalometric analysis methods (Figure 6). This subgroup also had the lowest inter-examiner reliability (Figure 7).

There is no doubt that this study had limitations. First, the CBCT datasets used in the current study did not have craniofacial syndromes or significant skeletal asymmetries. In a scenario with significant facial asymmetry, different borders of left- and right-side craniofacial structures may be distinguished on the extracted 2D lateral cephalometric X-rays even when using the setting of “orthogonal.” Thus, whether 2D tracings without magnification have higher reliability than tracings with conventional lateral cephalometric images for such patients’ needs to be verified. Additionally, only two American Board of Orthodontics certified clinicians were evaluated as examiners in the current study. The tracing performance of less experienced postgraduate program trainees in all three types of cephalometric analysis methods is worth considering to guide future clinical education and training.

We would like to emphasize that the CBCT datasets used in the current study were obtained from a pre-existing database of the patients who were prescribed with a full-volume CBCT for the initial purpose of evaluating impacted tooth/teeth, temporary anchorage device placement, orthognathic-orthodontic treatment plan, periodontal lesion, or endodontic lesion based on the clinical observation during the initial orthodontic consultation. In other words, no participants had a full-volume CBCT taken solely to extract lateral cephalometric images. We believe that all the radiological images in the orthodontic field should be taken with strict adherence to the ALARA (as low as reasonably achievable) principle. It is worth noting that, worldwide, substantial efforts have gone into further reducing radiation to the patients who need radiographic evaluation for orthodontic purposes. For example, a biplanar low-dose X-ray imaging system has been developed to take anteroposterior and lateral 2D images simultaneously, which can be used for 3D reconstruction based on statistical models [42]. Primarily used in the orthopedic field, this biplanar low-dose X-ray imaging system is capable of reliable cephalometric analysis [43]. Thus, for the patients who already have biplanar images for orthopedic purposes, no additional regular lateral/posterior-anterior cephalometric X-rays are needed if the patients are also seeking orthodontic and orthognathic management [43]. In addition, an ultra-low-dose CBCT imaging system has also been introduced in the dental field [44,45]. Excitingly, outside the range of 2 mm or degrees, there is no statistical difference in performing cephalometric analysis on the full-volume CBCTs taken under the ultra-low-dose protocol and standard protocol [38,45]. However, it has been noted that measurements based on images taken with the standard protocol have significantly smaller standard deviations than those taken with the ultra-low-dose protocol [38]. Moreover, previous studies indicate the patient scanning positions influence the accuracy of 3D cephalometry analysis, which could be sufficient to attract clinical attention [46,47]. Taken together, under current circumstances, commercially available 3D imaging systems cannot completely replace the 2D imaging systems regarding radiation exposure and analysis accuracy, and a large field CBCT scan for the sole reason of extracting a 2D pseudo-teleradiographic image is not good practice at the moment.

## 5. Conclusions

In summary, all three types of cephalometric analysis methods were reliable, with 2D tracings of extracted lateral cephalometric images without magnification having the highest intra- and inter-examiner reliabilities. However, since the current cephalometric norms were established using conventional lateral cephalometric images with built-in magnifications and distortions, 2D tracings of extracted lateral cephalometric images without magnification may not directly replace conventional cephalometric analysis. Further studies are needed to compare the tracing values between these two cephalometric analysis methods. Additionally, new landmarks are needed for 3D cephalometric tracing to improve the reliability of the 3D cephalometric analysis. Along with magnification, types of measurement parameters and patients’ ages are also influential factors in the accuracy and reliability of the cephalometric analysis. Clinical attention is needed when interpreting the angular measurements of images from children patients. Last but not least, the radiological images in the orthodontic field should be taken by strictly following the ALARA principle. Exposing a large field CBCT scan for the sole reason of extracting a 2D pseudo-teleradiographic image is not recommended.

## Figures and Tables

**Table 1:** The intra-examiner ICCs for each parameter. In each tracing scenario, the five parameters with the highest ICCs are labeled in green, and the five parameters with the lowest ICCs are marked in red.

Cephalometric Tracing Measurement Parameters		Examiner 1									Examiner 2								
		2D with Magnification			2D without Magnification			3D			2D with Magnification			2D without magnification			3D		
		ICC	95% CI Lower Bound	95% CI Upper Bound	ICC	95% CI Lower Bound	95% CI Upper Bound	ICC	95% CI Lower Bound	95% CI Upper Bound	ICC	95% CI Lower Bound	95% CI Upper Bound	ICC	95% CI Lower Bound	95% CI Upper Bound	ICC	95% CI Lower Bound	95% CI Upper Bound
Linear	B to A point arch (mm)	0.988	0.970	0.995	0.985	0.954	0.995	0.990	0.974	0.996	0.931	0.784	0.975	0.920	0.810	0.967	0.750	0.472	0.893
	Convexity (A-NPo) (mm)	0.978	0.945	0.991	0.990	0.973	0.996	0.988	0.970	0.995	0.948	0.859	0.980	0.847	0.654	0.936	0.848	0.656	0.937
	Corpus length (Go-Gn) (mm)	0.989	0.971	0.995	0.980	0.946	0.992	0.993	0.983	0.997	0.959	0.901	0.984	0.876	0.709	0.949	0.900	0.768	0.959
	Cranial length (mm)	0.944	0.864	0.978	0.905	0.780	0.961	0.917	0.795	0.967	0.816	0.590	0.923	0.837	0.635	0.932	0.696	0.378	0.867
	L1-APo (mm)	0.983	0.957	0.993	0.991	0.979	0.997	0.987	0.967	0.995	0.976	0.940	0.990	0.792	0.549	0.912	0.886	0.733	0.953
	L1-NB (mm)	0.980	0.949	0.992	0.978	0.907	0.993	0.993	0.981	0.997	0.820	0.603	0.924	0.796	0.559	0.913	0.852	0.669	0.938
	LAFH (ANS-Me) (mm)	0.998	0.994	0.999	0.997	0.991	0.999	0.993	0.984	0.997	0.868	0.699	0.946	0.864	0.693	0.944	0.945	0.866	0.978
	Lower Lip to E-Plane (mm)	0.986	0.964	0.994	0.984	0.960	0.993	0.991	0.978	0.997	0.935	0.845	0.973	0.730	0.439	0.883	0.704	0.391	0.871
	Mand. Skeletal (Pg-Na Perp) (mm)	0.948	0.873	0.979	0.991	0.977	0.996	0.973	0.934	0.989	0.894	0.753	0.957	0.934	0.794	0.976	0.942	0.861	0.977
	Mandibular Incisor Extrusion (mm)	0.953	0.885	0.981	0.960	0.904	0.984	0.961	0.903	0.984	0.686	0.356	0.863	0.925	0.824	0.969	0.379	−0.080	0.701
	Mandibular length (Co-Gn) (mm)	0.992	0.968	0.997	0.997	0.994	0.999	0.998	0.996	0.999	0.893	0.749	0.956	0.908	0.786	0.962	0.975	0.936	0.990
	Maxillary skeletal (A-Na Perp) (mm)	0.901	0.768	0.960	0.977	0.938	0.991	0.952	0.883	0.980	0.701	0.394	0.869	0.764	0.462	0.903	0.797	0.562	0.914
	Midface Length (Co-A) (mm)	0.971	0.878	0.990	0.987	0.967	0.995	0.989	0.972	0.996	0.813	0.584	0.922	0.940	0.854	0.976	0.864	0.687	0.944
	Molar Relation (mm)	0.966	0.916	0.986	0.942	0.860	0.977	0.957	0.894	0.983	0.687	0.361	0.863	0.922	0.816	0.969	0.899	0.764	0.959
	Mx/Md diff (Co-Gn-Co-A) (mm)	0.995	0.988	0.998	0.995	0.987	0.998	0.995	0.987	0.998	0.974	0.929	0.990	0.890	0.744	0.955	0.961	0.892	0.985
	Overbite (mm)	0.952	0.883	0.981	0.963	0.910	0.985	0.963	0.909	0.985	0.785	0.539	0.909	0.926	0.825	0.970	0.582	0.207	0.810
	Overjet (mm)	0.973	0.933	0.989	0.993	0.984	0.997	0.994	0.984	0.998	0.706	0.401	0.871	0.942	0.861	0.976	0.859	0.678	0.942
	Pg to ANS arc (mm)	0.968	0.920	0.987	0.986	0.964	0.994	0.969	0.923	0.988	0.860	0.684	0.942	0.903	0.728	0.963	0.694	0.382	0.866
	Pog-NB (mm)	0.903	0.772	0.961	0.933	0.617	0.980	0.900	0.765	0.959	0.837	0.639	0.932	0.839	0.637	0.933	0.769	0.501	0.902
	Porion location (mm)	0.915	0.799	0.966	0.960	0.901	0.984	0.947	0.873	0.979	0.828	0.620	0.928	0.699	0.386	0.869	0.845	0.638	0.936
	Posterior facial height (Go-CF) (mm)	0.992	0.981	0.997	0.994	0.983	0.998	0.996	0.988	0.998	0.969	0.925	0.988	0.899	0.762	0.959	0.982	0.951	0.993
	U1 most labial-A (perp to FH) (mm)	0.983	0.957	0.993	0.989	0.972	0.996	0.992	0.980	0.997	0.962	0.903	0.985	0.764	0.496	0.899	0.857	0.674	0.941
	U1 to ANS arc (mm)	0.969	0.924	0.987	0.979	0.949	0.992	0.967	0.918	0.987	0.887	0.741	0.953	0.854	0.539	0.948	0.756	0.477	0.896
	U1-APo (mm)	0.976	0.941	0.990	0.997	0.992	0.999	0.990	0.976	0.996	0.851	0.666	0.938	0.832	0.622	0.930	0.908	0.784	0.962
	U1-NA (mm)	0.983	0.958	0.993	0.993	0.983	0.997	0.986	0.965	0.994	0.927	0.825	0.971	0.765	0.494	0.900	0.821	0.600	0.925
	U6-PT vertical (mm)	0.977	0.944	0.991	0.970	0.927	0.988	0.971	0.930	0.988	0.811	0.589	0.920	0.812	0.499	0.928	0.841	0.631	0.935
	Upper Lip to E-Plane (mm)	0.976	0.941	0.990	0.973	0.932	0.989	0.952	0.883	0.981	0.954	0.887	0.982	0.787	0.534	0.910	0.867	0.684	0.946
	Wits Appraisal (mm)	0.978	0.946	0.991	0.976	0.936	0.991	0.965	0.914	0.986	0.960	0.903	0.984	0.928	0.831	0.971	0.789	0.537	0.911
Angular	ANB (°)	0.973	0.934	0.989	0.986	0.966	0.994	0.985	0.963	0.994	0.953	0.887	0.981	0.886	0.733	0.953	0.785	0.541	0.908
	Cranial deflection (°)	0.616	0.244	0.829	0.971	0.925	0.989	0.914	0.798	0.965	0.651	0.311	0.845	0.736	0.328	0.897	0.767	0.499	0.901
	Facial angle (FH/NPo) (°)	0.947	0.871	0.979	0.990	0.974	0.996	0.971	0.930	0.988	0.701	0.295	0.869	0.854	0.295	0.949	0.920	0.811	0.968
	Facial axis angle (Ba-Na^Pt-Gn) (°)	0.963	0.910	0.985	0.986	0.965	0.994	0.993	0.983	0.997	0.977	0.943	0.991	0.924	0.818	0.969	0.928	0.829	0.971
	Facial Axis-Ricketts (NaBa-PtGn) (°)	0.963	0.910	0.985	0.986	0.965	0.994	0.993	0.983	0.997	0.677	0.355	0.857	0.921	0.813	0.968	0.924	0.821	0.969
	Facial taper (°)	0.982	0.954	0.993	0.981	0.946	0.993	0.991	0.977	0.996	0.920	0.808	0.967	0.845	0.654	0.935	0.859	0.684	0.941
	FMA (MP-FH) (°)	0.938	0.851	0.975	0.979	0.928	0.993	0.982	0.956	0.993	0.910	0.789	0.963	0.902	0.690	0.965	0.953	0.886	0.981
	FMIA (L1-FH) (°)	0.940	0.856	0.967	0.963	0.893	0.986	0.961	0.905	0.985	0.846	0.655	0.936	0.841	0.627	0.935	0.850	0.664	0.938
	GoGn to FH (FMA) (°)	0.924	0.821	0.969	0.976	0.940	0.990	0.982	0.956	0.993	0.886	0.738	0.953	0.796	0.406	0.925	0.917	0.803	0.966
	Hinge Axis Angle (°)	0.941	0.858	0.976	0.959	0.896	0.984	0.949	0.876	0.979	0.883	0.731	0.952	0.757	0.478	0.897	0.744	0.465	0.890
	IMPA (L1-MP) (°)	0.954	0.887	0.981	0.961	0.906	0.984	0.932	0.837	0.973	0.872	0.704	0.947	0.859	0.679	0.942	0.762	0.487	0.899
	Interincisal Angle (U1-L1) (°)	0.955	0.890	0.982	0.975	0.940	0.990	0.961	0.907	0.984	0.914	0.795	0.965	0.795	0.558	0.913	0.723	0.425	0.880
	L1-APo (°)	0.975	0.928	0.990	0.961	0.891	0.985	0.940	0.856	0.976	0.932	0.822	0.973	0.799	0.556	0.915	0.811	0.587	0.921
	L1-NB (°)	0.958	0.898	0.983	0.957	0.894	0.983	0.932	0.837	0.973	0.848	0.655	0.937	0.801	0.562	0.917	0.743	0.466	0.889
	Lower face height (ANS-Xi-Pm) (°)	0.989	0.971	0.995	0.991	0.977	0.996	0.993	0.983	0.997	0.887	0.740	0.953	0.909	0.769	0.964	0.948	0.873	0.979
	Lower Gonial angle (Na-Go-Me) (°)	0.982	0.995	0.993	0.989	0.973	0.996	0.994	0.986	0.998	0.828	0.619	0.928	0.908	0.785	0.962	0.917	0.802	0.966
	Mandibular Arc (°)	0.946	0.869	0.978	0.898	0.766	0.958	0.963	0.909	0.985	0.762	0.481	0.900	0.648	0.302	0.844	0.697	0.382	0.867
	Maxillary depth (FH-NA) (°)	0.970	0.925	0.988	0.979	0.945	0.992	0.952	0.885	0.981	0.808	0.579	0.919	0.765	0.448	0.905	0.972	0.933	0.989
	Maxillary height (N-CF-A) (°)	0.956	0.895	0.982	0.965	0.914	0.986	0.967	0.919	0.987	0.878	0.723	0.950	0.844	0.628	0.937	0.880	0.720	0.951
	MP-SN (°)	0.987	0.967	0.995	0.987	0.969	0.995	0.991	0.977	0.996	0.961	0.905	0.984	0.877	0.719	0.949	0.916	0.802	0.966
	Occlusal plane to FH (°)	0.942	0.862	0.977	0.949	0.741	0.984	0.971	0.929	0.988	0.811	0.584	0.920	0.875	0.697	0.950	0.760	0.496	0.897
	Occlusal Plane to SN (°)	0.964	0.913	0.985	0.973	0.925	0.990	0.966	0.916	0.986	0.946	0.866	0.978	0.936	0.846	0.974	0.807	0.572	0.919
	Ramus position (°)	0.806	0.570	0.919	0.934	0.841	0.973	0.960	0.899	0.984	0.607	0.247	0.822	0.488	0.034	0.768	0.847	0.623	0.939
	SN-palatal plane (°)	0.906	0.780	0.961	0.839	0.637	0.933	0.979	0.948	0.992	0.830	0.619	0.929	0.798	0.554	0.915	0.546	0.168	0.789
	SNA (°)	0.980	0.950	0.992	0.988	0.971	0.995	0.991	0.978	0.997	0.871	0.708	0.947	0.906	0.777	0.962	0.876	0.671	0.952
	SNB (°)	0.991	0.977	0.996	0.988	0.971	0.995	0.990	0.976	0.996	0.842	0.635	0.935	0.939	0.853	0.975	0.945	0.867	0.978
	Soft tissue Convexity (°)	0.968	0.921	0.987	0.932	0.837	0.973	0.870	0.707	0.946	0.796	0.557	0.914	0.900	0.770	0.959	0.589	0.199	0.815
	Total face height (NaBa-PmXi) (°)	0.965	0.915	0.986	0.988	0.970	0.995	0.995	0.986	0.998	0.887	0.737	0.954	0.918	0.808	0.967	0.949	0.878	0.979
	U1- Palatal Plane (°)	0.940	0.856	0.976	0.946	0.865	0.979	0.958	0.896	0.983	0.810	0.581	0.921	0.688	0.373	0.863	0.781	0.522	0.907
	U1-APo (°)	0.905	0.775	0.961	0.920	0.729	0.972	0.926	0.822	0.970	0.748	0.461	0.892	0.784	0.525	0.909	0.623	0.263	0.832
	U1-FH (°)	0.947	0.871	0.979	0.950	0.832	0.982	0.968	0.920	0.987	0.841	0.641	0.934	0.779	0.373	0.918	0.828	0.622	0.928
	U1-NA (°)	0.959	0.900	0.984	0.964	0.884	0.987	0.958	0.898	0.983	0.913	0.796	0.964	0.776	0.524	0.904	0.857	0.678	0.940
	U1-SN (°)	0.959	0.899	0.983	0.967	0.899	0.988	0.973	0.933	0.989	0.883	0.728	0.952	0.851	0.657	0.939	0.845	0.654	0.935
	Upper gonial angle (Ar-Go-Na) (°)	0.972	0.930	0.989	0.990	0.974	0.996	0.992	0.980	0.997	0.961	0.907	0.984	0.895	0.727	0.959	0.944	0.866	0.977
	Upper lip angle (ULA) (°)	0.970	0.925	0.988	0.970	0.925	0.988	0.959	0.900	0.984	0.923	0.794	0.970	0.892	0.749	0.956	0.923	0.816	0.969

**Table 2:** The inter-examiner ICCs for each parameter. In each tracing scenario, the five parameters with the highest ICCs are labeled in green, and the five parameters with the lowest ICCs are marked in red.

Cephalometric Tracing Measurement Parameters		Inter-Examiner								
		2D with Magnification			2D without Magnification			3D		
		ICC	95% CI Lower Bound	95% CI Upper Bound	ICC	95% CI Lower Bound	95% CI Upper Bound	ICC	95% CI Lower Bound	95% CI Upper Bound
Linear	B to A point arch (mm)	0.960	0.887	0.985	0.957	0.839	0.985	0.653	0.136	0.866
	convexity (A-NPo) (mm)	0.965	0.911	0.986	0.928	0.819	0.971	0.599	0.043	0.844
	Corpus length (Go-Gn) (mm)	0.862	0.689	0.943	0.961	0.906	0.984	0.940	0.857	0.976
	cranial length (mm)	0.872	0.709	0.947	0.875	0.492	0.959	0.416	−0.051	0.730
	L1-APo (mm)	0.839	0.642	0.933	0.928	0.828	0.971	0.718	0.423	0.877
	L1-NB (mm)	0.763	0.500	0.898	0.939	0.853	0.975	0.776	0.519	0.905
	LAFH (ANS-Me) (mm)	0.824	0.611	0.926	0.937	0.837	0.975	0.793	0.555	0.912
	Lower Lip to E-Plane (mm)	0.748	0.453	0.894	0.891	0.734	0.956	0.606	0.113	0.840
	Mand. Skeletal (Pg-Na Perp) (mm)	0.925	0.786	0.972	0.942	0.860	0.977	0.934	0.841	0.973
	Mandibular Incisor Extrusion (mm)	0.758	0.481	0.897	0.868	0.697	0.946	0.609	0.216	0.828
	Mandibular length (Co-Gn) (mm)	0.927	0.826	0.971	0.979	0.948	0.992	0.928	0.831	0.971
	Maxillary skeletal (A-Na Perp) (mm)	0.778	0.520	0.906	0.871	0.706	0.947	0.678	0.230	0.871
	Midface Length (Co-A) (mm)	0.890	0.746	0.955	0.978	0.946	0.991	0.843	0.465	0.945
	Molar Relation (mm)	0.884	0.734	0.952	0.957	0.896	0.983	0.918	0.807	0.967
	Mx/Md diff (Co-Gn-Co-A) (mm)	0.939	0.853	0.975	0.982	0.955	0.993	0.902	0.770	0.960
	Overbite (mm)	0.790	0.539	0.911	0.865	0.691	0.945	0.701	0.320	0.877
	Overjet (mm)	0.907	0.780	0.962	0.961	0.906	0.984	0.852	0.669	0.938
	Pg to ANS arc (mm)	0.691	−0.052	0.906	0.688	−0.016	0.900	0.829	0.616	0.929
	Pog-NB (mm)	0.919	0.772	0.969	0.900	0.742	0.961	0.802	0.563	0.917
	porion location (mm)	0.748	0.462	0.892	0.894	0.755	0.956	0.562	−0.098	0.855
	posterior facial height (Go-CF) (mm)	0.928	0.830	0.971	0.955	0.784	0.986	0.915	0.799	0.965
	U1 most labial-A (perp to FH) (mm)	0.859	0.683	0.942	0.903	0.772	0.960	0.705	0.347	0.876
	U1 to ANS arc (mm)	0.464	−0.104	0.792	0.489	−0.102	0.809	0.753	0.481	0.894
	U1-APo (mm)	0.807	0.579	0.919	0.946	0.870	0.987	0.786	0.534	0.909
	U1-NA (mm)	0.822	0.730	0.952	0.917	0.806	0.966	0.720	0.288	0.892
	U6-PT vertical (mm)	0.821	0.608	0.925	0.909	0.683	0.968	0.788	0.537	0.911
	Upper Lip to E-Plane (mm)	0.798	0.465	0.922	0.834	0.568	0.935	0.697	0.205	0.885
	Wits Appraisal (mm)	0.938	0.850	0.975	0.936	0.846	0.974	0.742	0.299	0.903
Angular	ANB (°)	0.967	0.919	0.987	0.918	0.807	0.966	0.574	−0.042	0.843
	cranial deflection (°)	0.589	0.072	0.834	0.771	0.506	0.903	0.764	0.490	0.900
	facial angle (FH/NPo) (°)	0.830	0.541	0.935	0.917	0.799	0.967	0.939	0.854	0.976
	Facial axis angle (Ba-Na^Pt-Gn) (°)	0.905	0.776	0.961	0.961	0.905	0.984	0.893	0.529	0.966
	Facial Axis-Ricketts (NaBa-PtGn) (°)	0.703	0.389	0.871	0.961	0.907	0.984	0.890	0.503	0.965
	Facial taper (°)	0.907	0.783	0.962	0.885	0.665	0.957	0.853	0.630	0.942
	FMA (MP-FH) (°)	0.782	0.524	0.908	0.888	0.735	0.955	0.853	0.666	0.939
	FMIA (L1-FH) (°)	0.815	0.597	0.922	0.805	0.577	0.917	0.679	0.343	0.860
	GoGn to FH (FMA) (°)	0.931	0.837	0.972	0.897	0.761	0.958	0.861	0.686	0.942
	Hinge Axis Angle (°)	0.801	0.522	0.920	0.783	0.503	0.910	0.675	0.345	0.857
	IMPA (L1-MP) (°)	0.847	0.642	0.938	0.829	0.623	0.928	0.658	0.323	0.848
	Interincisal Angle (U1-L1) (°)	0.750	0.451	0.895	0.857	0.608	0.945	0.632	0.281	0.835
	L1-APo (°)	0.824	0.601	0.927	0.835	0.584	0.935	0.477	0.072	0.751
	L1-NB (°)	0.754	0.478	0.895	0.817	0.591	0.923	0.542	0.132	0.791
	Lower face height (ANS-Xi-Pm) (°)	0.807	0.466	0.927	0.899	0.673	0.964	0.828	0.615	0.928
	Lower Gonial angle (Na-Go-Me) (°)	0.922	0.814	0.968	0.914	0.789	0.965	0.837	0.618	0.933
	Mandibular Arc (°)	0.680	−0.027	0.898	0.683	0.232	0.875	0.864	0.692	0.943
	maxillary depth (FH-NA) (°)	0.469	0.068	0.745	0.887	0.740	0.954	0.701	0.268	0.882
	maxillary height (N-CF-A) (°)	0.769	0.465	0.905	0.866	0.566	0.952	0.822	0.595	0.926
	MP-SN (°)	0.905	0.776	0.961	0.911	0.767	0.966	0.823	0.522	0.932
	Occlusal plane to FH (°)	0.733	0.366	0.893	0.786	0.533	0.910	0.696	0.384	0.867
	Occlusal Plane to SN (°)	0.771	0.434	0.909	0.931	0.837	0.972	0.881	0.644	0.956
	ramus position (°)	0.611	0.256	0.823	0.723	0.428	0.879	0.654	0.253	0.854
	SN-palatal plane (°)	0.890	0.747	0.955	0.847	0.610	0.940	0.791	0.547	0.911
	SNA (°)	0.939	0.822	0.977	0.937	0.848	0.975	0.780	0.458	0.913
	SNB (°)	0.953	0.843	0.983	0.974	0.935	0.989	0.951	0.866	0.981
	Soft tissue Convexity (°)	0.905	0.779	0.961	0.937	0.848	0.975	0.515	0.111	0.774
	Total face height (NaBa-PmXi) (°)	0.927	0.826	0.971	0.937	0.850	0.740	0.957	0.895	0.983
	U1- Palatal Plane (°)	0.707	0.349	0.878	0.861	0.518	0.952	0.710	0.250	0.889
	U1-APo (°)	0.586	0.221	0.811	0.851	0.667	0.938	0.632	0.276	0.836
	U1-FH (°)	0.731	0.382	0.889	0.850	0.608	0.942	0.867	0.667	0.947
	U1-NA (°)	0.788	0.539	0.910	0.871	0.673	0.949	0.755	0.079	0.923
	U1-SN (°)	0.806	0.529	0.922	0.920	0.804	0.968	0.887	0.500	0.964
	upper gonial angle (Ar-Go-Na) (°)	0.917	0.798	0.967	0.965	0.804	0.989	0.921	0.295	0.977
	Upper lip angle (ULA) (°)	0.875	0.610	0.955	0.956	0.864	0.984	0.768	0.509	0.901

## Data Availability

The data presented in this study are contained within this article and Supplementary Materials.

## Supplementary Materials

The following are available online at https://www.mdpi.com/article/10.3390/diagnostics11122292/s1, Supplemental Table S1: The intra-examiner intraclass correlation coefficient (ICC) of examiner 1 of all measurement parameters for the radiographic images from different age groups of the patients; Supplemental Table S2: The intra-examiner intraclass correlation coefficient (ICC) of examiner 2 of all measurement parameters for the radiographic images from different age groups of the patients; Supplemental Table S3: The inter-examiner intraclass correlation coefficient (ICC) of all measurement parameters for the radiographic images from different age groups of the patients.
